# EasyModel: a user-friendly web-based interface based on MODELLER

**DOI:** 10.1038/s41598-023-44505-9

**Published:** 2023-10-11

**Authors:** Seyed Shahriar Arab, Alireza Dantism

**Affiliations:** Department of Biophysics, Faculty of Biological Sciences, Tarbiat Modares University, 1411713116 Terhan, Iran

**Keywords:** Computational biology and bioinformatics, Structural biology

## Abstract

Three-dimensional protein structures are invaluable sources of information for the functional annotation of protein molecules. Describing the function of a protein sequence is one of the most common problems in biology. Generally, this problem can be facilitated by studying the tertiary structure of proteins. In the lack of protein structures, comparative modeling often provides a useful three-dimensional model of the protein associated with at least one known protein structure. Comparative modeling predicts the tertiary structure of a certain protein sequence (target) mainly based on its homological sequence to the sequence of one or more proteins with known structures (templates). MODELLER is one of the most widely used tools for homology or comparative modeling of three-dimensional protein structures. However, most users find it challenging to start with MODELLER as it is a command line based and requires knowledge of basic Python scripting to use it efficiently. In this study, a web-based interface has been designed to predict the tertiary structure of proteins based on Modeller, which does the comparative modeling automatically, and uses PHP and Python programming languages. This tool is called “EasyModel” and is available at http://bioinf.modares.ac.ir/software/easymodel/. EasyModel provides a straightforward graphical interface for Modeller that can be used in only one browser.

## Introduction

The basis of prediction by homology modeling is that the protein sequence is similar to one or more proteins with known structures. Based on the fact that proteins with similar sequences have identical structures. Homology modeling predicts a given protein sequence (target) establish on its alignment with one or more proteins of known structure (templates) and the alignment of target and template(s) sequences.

The steps of this process are: (1) identification of homologs that can serve as template(s) for modeling; (2) alignment of the target sequence to the template(s); (3) backbone generation; (4) loop modeling; (5) side-chain modeling; (6) model optimization; and (7) validation of the model^1^.

Several computer programs and web servers serve as a graphical user interface for Modeller^2^. These interfaces are categorized into two types, web-based and desktop applications.

Among the web-based applications, Esypred3D^3^, HHpred^4^, and Modbase^5^ can be mentioned. For desktop applications, ChimeraX^6^, and EasyModeller^7^ are notable choices.

Modeller is a widely used program for the homology modeling of proteins, though some users find it challenging to navigate due to its command line interface and reliance on Python expertise. As a command line application without a graphical interface, users must possess basic knowledge of both the command line and Python to achieve optimal performance. To address this issue, various companies and individuals have developed visual interface software to make Modeller more accessible to users. However, these interfaces are depending on the operating system (Windows or Mac) or they consider it necessary to install Python on the operating system, also, some of them are no longer available or not under active development. Most of these programs are limited to basic modeling, and many older programs are no longer available due to their incompatibility with newer versions of Modeller.

Therefore, the approach adopted in this study is developing and implementing an online bioinformatics interface for predicting the tertiary structure of proteins. For this prediction, the homology modeling method and Modeller are used. All the parts that researchers required, including Python, and the command line, have been removed so that researchers can complete all their modeling steps with only a straightforward graphical interface.

The main focus of this study is to create an online bioinformatics tool that can predict the tertiary structure of proteins. To achieve this goal, the homology modeling method and Modeller are utilized. The tool has been designed to offer a simple graphical interface, eliminating the need for researchers to have experience with Python scripts or the command line. This simplifies the modeling process, allowing researchers to complete all necessary steps easily.

## Results

**Figure 1:**
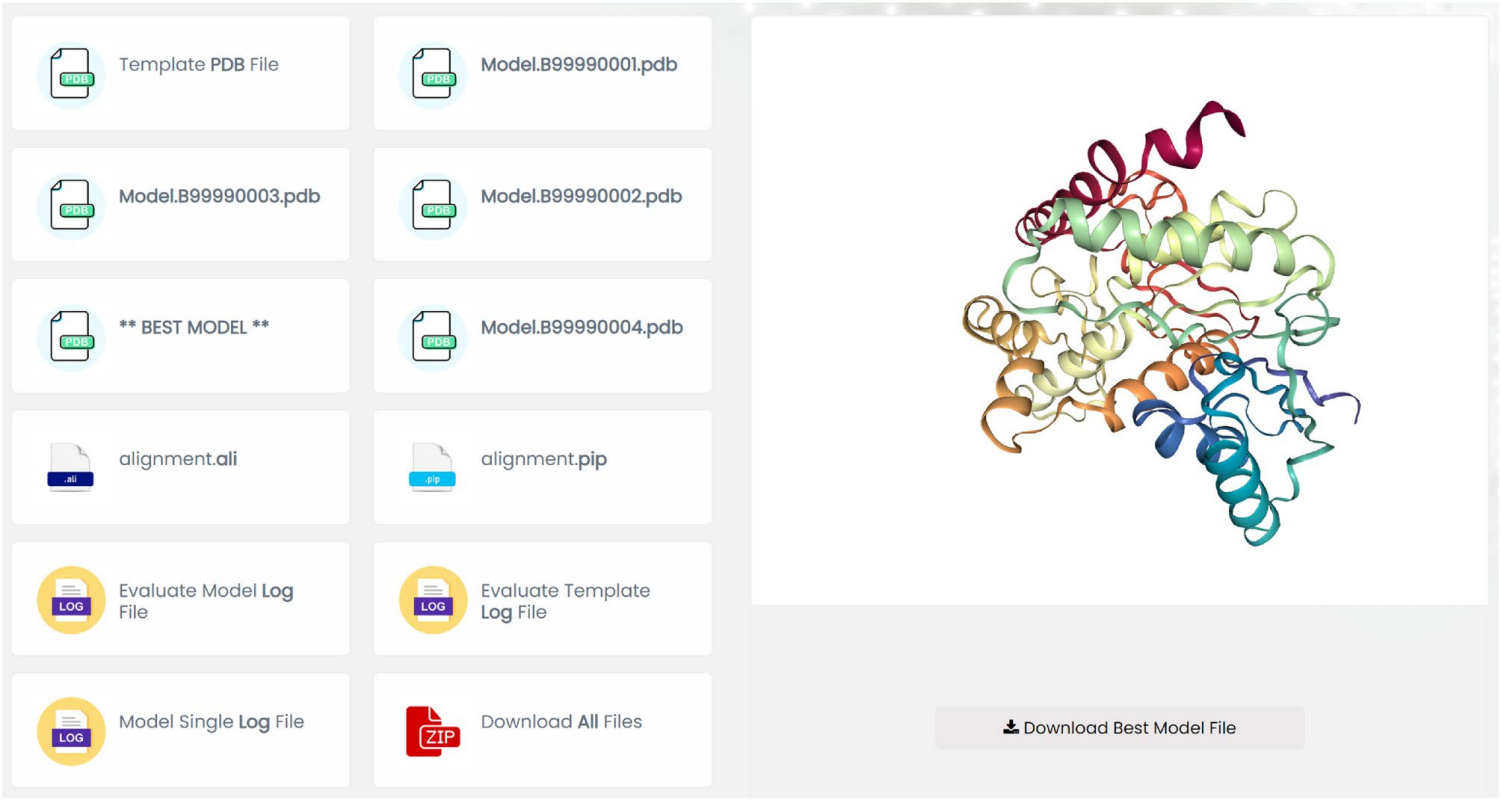
Show results after basic modeling and advanced modelling (loop refinement and multiple templates) in EasyModel.

We utilized the lactate dehydrogenase enzyme sequence as a sample for modeling purposes. Upon completing the initial modeling process, we obtained a diagram named the DOPE (Discrete Optimized Protein Energy)^8^ score diagram (Fig. 4), which displays the quality of the constructed model per residue in comparison with the target protein. Access to all data generated by the Modeler program is also provided through the provided link (Fig. 1).

The NGL viewer displays the best model on the right-hand side, while a list of available and downloadable models is provided on the left-hand side. The model with the lowest DOPE Score is also identified as the BEST MODEL. In addition to the built models, alignment files in ‘pir’ and ‘ali’ formats, as well as log files, can be downloaded individually or as a compressed file. To ensure the accuracy of the protein model, it is strongly advised to thoroughly review the sequence alignment. As such, the report includes an illustration of the sequence alignment at the end. All of these modeling tasks can be performed in just a few minutes, without requiring any programming or Python knowledge, or the installation of necessary software. The successful integration of the Modeller academic license into EasyModel’s server has enabled the user-friendly graphical interface for Modeller to be up and running. To make use of EasyModel, ensure you possess a valid Modeller academic license key, which can be obtained by visiting the website of Andrej Sali’s lab at https://salilab.org.

## Discussion

In addition to the results obtained from this study, exploring other capabilities of modeller, such as analyzing protein-ligand interactions and generating independent diagrams of protein profiles, can further aid scientists in predicting the tertiary structure of sequences.

## Methods

**Figure 2:**
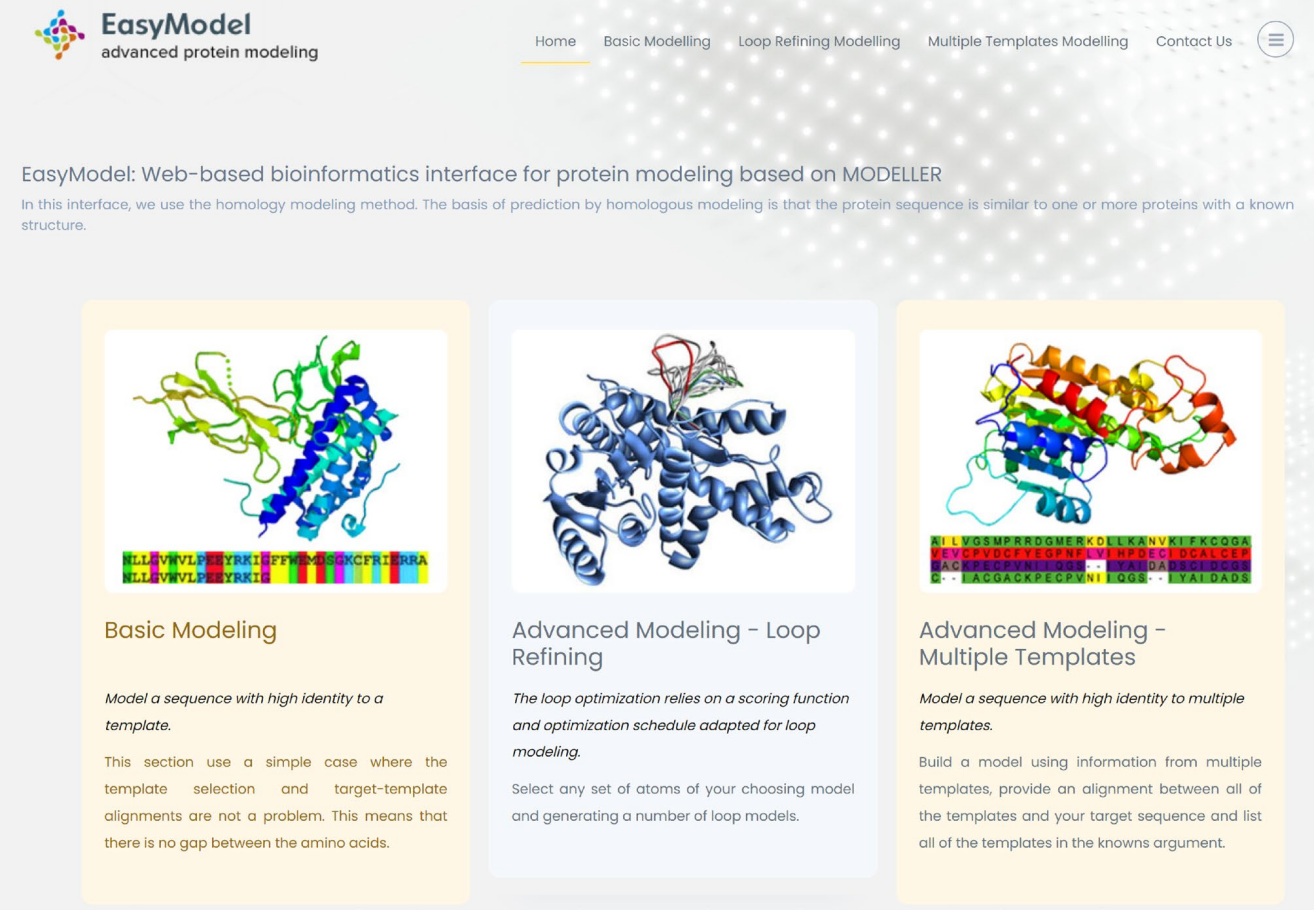
Screenshot of the EasyModel that consists of basic modelling, advanced modelling (loop refinement), and advanced modelling (multiple templates).

The primary objective of this study has been to create a frontend graphical user interface called “EasyModel” for Modeller, which has been built using PHP and Python. It is worth mentioning that the frontend of the tool has been developed using HTML5, Bootstrap, CSS, and JavaScript, furthermore, NGL viewer^9^ is used to display molecules. This web-based interface has been designed to assist users who lack programming skills, knowledge of the command line, and proficiency in the Python programming language. With EasyModel, users can easily model, evaluate, analyze, and optimize protein models. The interface is capable of identifying the target sequence and template(s) structure, predicting the tertiary structure of the target sequence, and presenting the results in the form of graphs, log files, alignments, and data in constructed protein files. The screenshot of the EasyModel interface is shown in Fig. 2.

EasyModel offers three different sections to predict the tertiary structures of proteins. Based on their specific requirements, users can select one of these sections. The three sections available are: (1) basic modeling. (2) Advanced modeling-loop refining. (3) Advanced modeling-multiple templates.

### Basic modelling

In cases where template selection and target-template alignments pose no challenge, basic modeling can be readily employed as a straightforward approach. Detailed step-by-step instructions on how to perform these steps are fully available at https://salilab.org/modeller/tutorial/basic.html.

**Figure 3:**
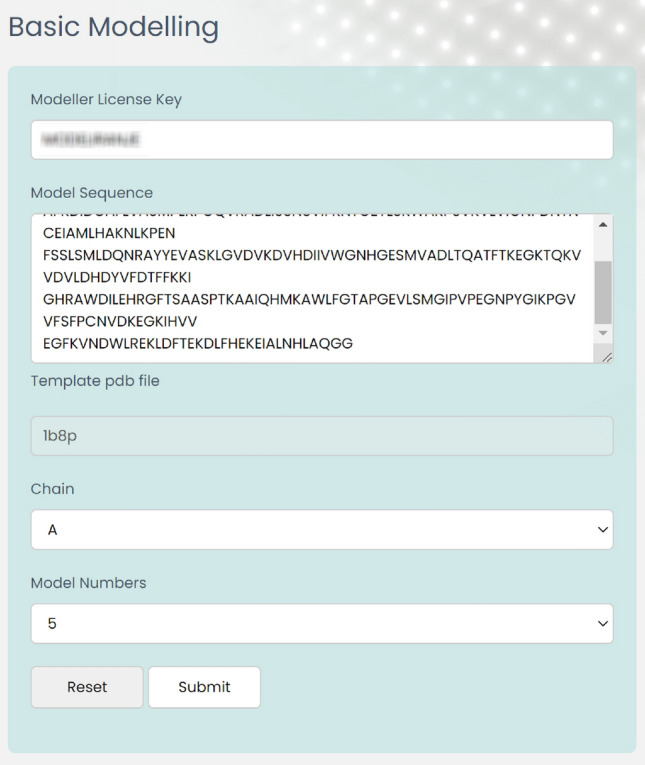
Screenshot of the basic modeling section in EasyModel.

**Figure 4:**
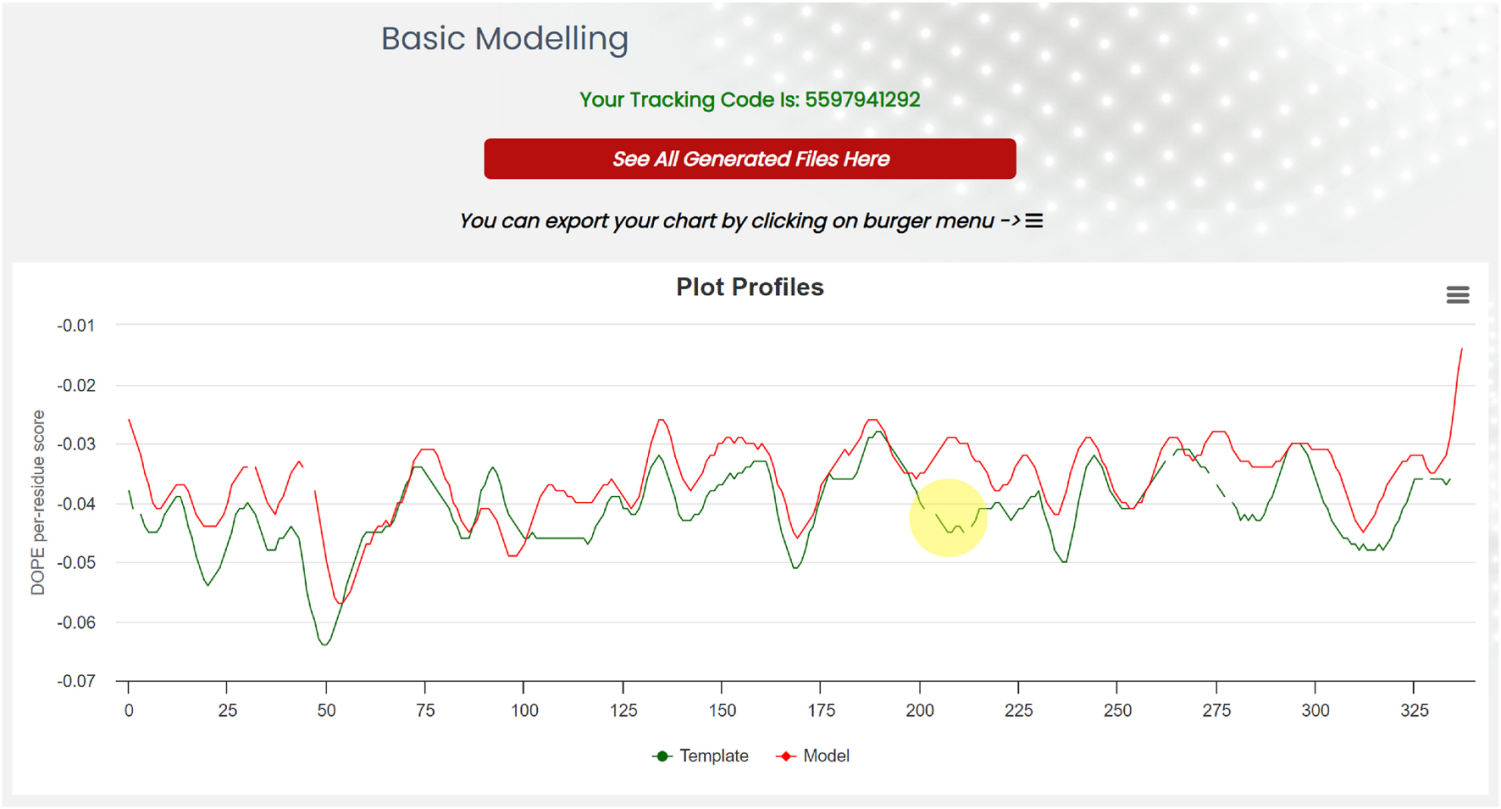
The DOPE scores for individual residues are depicted with green and red lines representing. The scores of the template and model structures, respectively. The yellow area indicates structural gaps in the template that have resulted in a poor DOPE score due to the absence of a modeling template in this region. These regions need to improve their modeling.

In the basic modeling section of the designed tool, users are able to import their desired protein PDB file. Here, the various steps of protein modeling using this tool for the lactate dehydrogenase protein sequence are demonstrated (Fig. 3, 4).

Upon basic modeling operations, EasyModel automatically generates a log file. Following the Basic Modeling step, a graph illustrating the DOPE scores of both the input template and the generated model is presented. The DOPE score is a statistical potential-based scoring function utilized to assess the quality of a protein structure model by estimating its relative energy. This score enables the comparison and ranking of different models, with lower scores indicating higher-quality protein structure models. The resulting chart can be downloaded in various file formats, including jpg, png, pdf, and others (Fig. 4).

In Fig. 4, the yellow area highlights structural gaps in the template, resulting in high DOPE scores due to the lack of a modeling template in this region. To address these regions, advanced modeling—loop modeling—will be employed to improve the model’s accuracy. By clicking on the “See All Generated Files” button, users are directed to the modeling results page. More details about the results will be provided in the results section. Additionally, you can find a sample result at http://bioinf.modares.ac.ir/software/easymodel/basic-modelling/sample.

### Advanced modelling—multiple templates

In protein modeling, multiple templates are employed when there is no single template that closely matches the target protein sequence. In such scenarios, various templates can be utilized to model different segments of the target protein sequence, and subsequently, these models can be combined to generate a final model. The utilization of multiple templates can enhance the accuracy of the resulting model, particularly when the target protein possesses distinct structural features that are absent in any of the individual templates.

**Figure 5:**
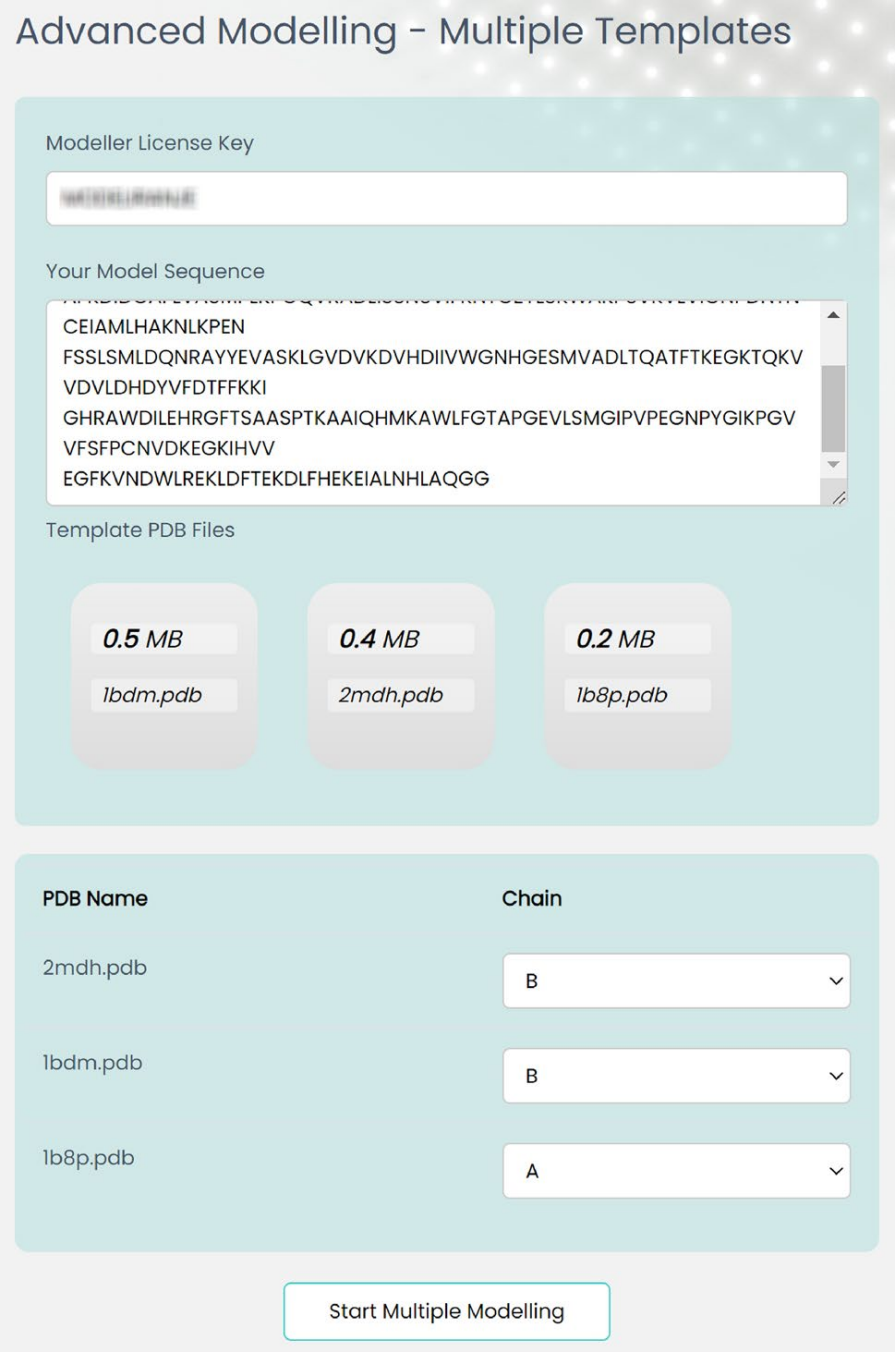
Advanced modeling with multiple templates in EasyModel.

As it is shown in Fig. 5, EasyModel then automatically starts to perform modeling operations with several models. After the completion of the modeling process, the results page will be presented, providing detailed information in the results section. You can also view a sample result at http://bioinf.modares.ac.ir/software/easymodel/advanced-modelling/multiple-templates/sample.

### Advanced modelling—loop refining

**Figure 6:**
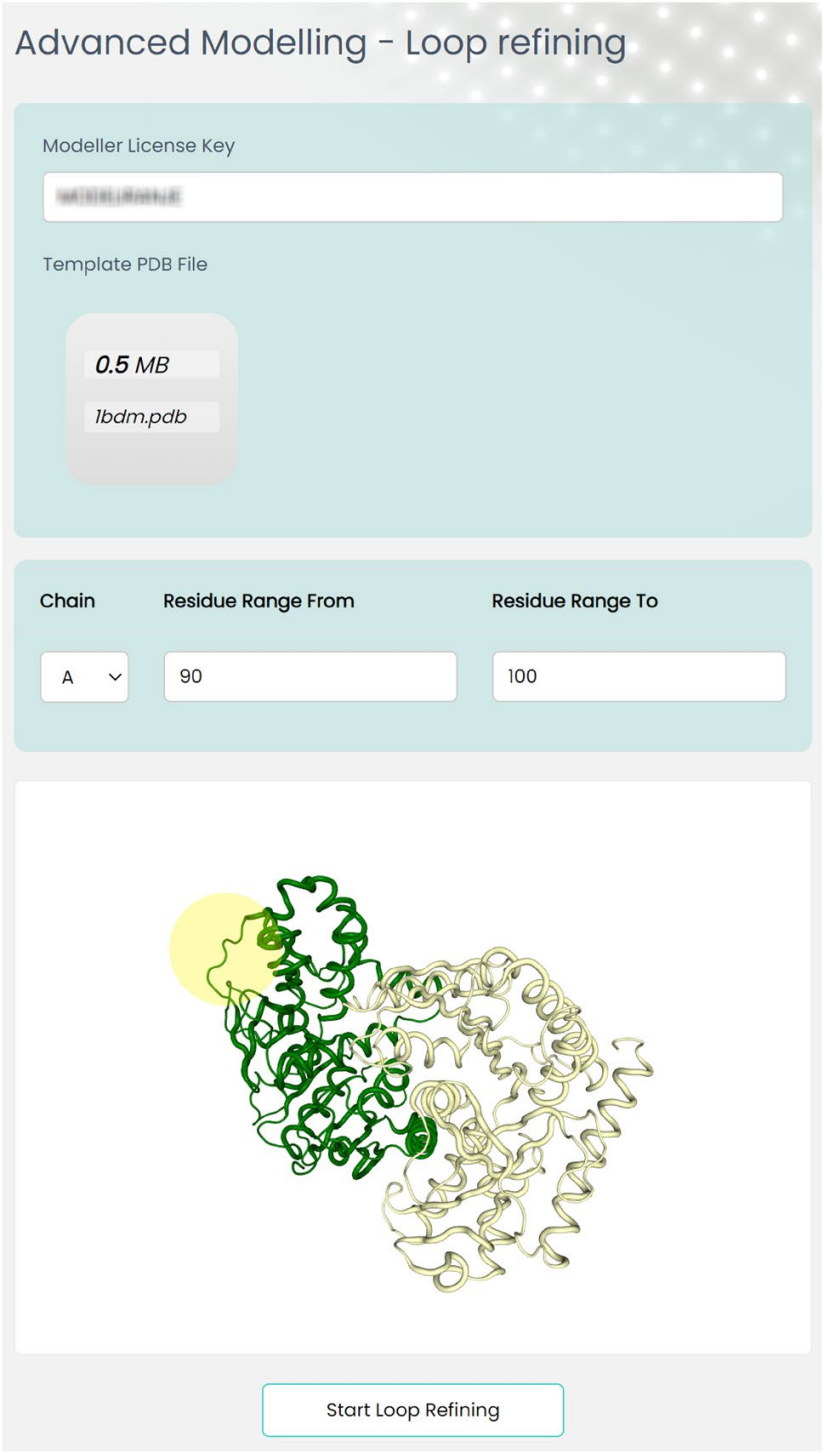
Selection of side chain and amino acid range in loop refining in EasyModel.

Loop Refining improves the accuracy of a protein structure model by optimizing poorly modeled loops. This method utilizes scoring functions and optimization protocols specially designed for loop modeling. It can also enhance the accuracy of a particular range of residues in the model^10^. Figure 6 demonstrates how to select the chain and the range of residues within a loop that requires refining after uploading the protein structure file.

Upon completing the Loop Refining process, 10 different models and a log file of the program execution are created and saved. You can explore a sample result page at http://bioinf.modares.ac.ir/software/easymodel/advanced-modelling/loop-refining/sample.

Comprehensive, detailed, and step-by-step instructions for performing Modeller advanced modeling steps are fully accessible at https://salilab.org/modeller/tutorial/advanced.html.

## Data Availability

EasyModel is available on Github, allowing users to access the source code, customize the interface to their requirements, and contribute to the development of EasyModel. Visit our GitHub repository athttps://github.com/alireza-dantism/EasyModel.
